# Fast and Slow Readers of the Hebrew Language Show Divergence in Brain Response ∼200 ms Post Stimulus: An ERP Study

**DOI:** 10.1371/journal.pone.0103139

**Published:** 2014-07-31

**Authors:** Sebastian Peter Korinth, Zvia Breznitz

**Affiliations:** 1 Goethe University Frankfurt am Main, Department of Neurocognitive Psychology, Frankfurt am Main, Germany; 2 Center for Individual Development and Adaptive Education of Children at Risk (IDeA), Frankfurt am Main, Germany; 3 Edmond J. Safra Brain Research Center for the Study of Learning Disabilities at the University of Haifa, Haifa, Israel; Birkbeck College, United Kingdom

## Abstract

Higher N170 amplitudes to words and to faces were recently reported for faster readers of German. Since the shallow German orthography allows phonological recoding of single letters, the reported speed advantages might have their origin in especially well-developed visual processing skills of faster readers. In contrast to German, adult readers of Hebrew are forced to process letter chunks up to whole words. This dependence on more complex visual processing might have created ceiling effects for this skill. Therefore, the current study examined whether also in the deep Hebrew orthography visual processing skills as reflected by N170 amplitudes explain reading speed differences. Forty university students, native speakers of Hebrew without reading impairments, accomplished a lexical decision task (i.e., deciding whether a visually presented stimulus represents a real or a pseudo word) and a face decision task (i.e., deciding whether a face was presented complete or with missing facial features) while their electroencephalogram was recorded from 64 scalp positions. In both tasks stronger event related potentials (ERPs) were observed for faster readers in time windows at about 200 ms. Unlike in previous studies, ERP waveforms in relevant time windows did not correspond to N170 scalp topographies. The results support the notion of visual processing ability as an orthography independent marker of reading proficiency, which advances our understanding about regular and impaired reading development.

## Introduction

Most present day work situations demand high levels of fast and accurate text processing skills. However, in research there is not a clear answer yet, why within the normal range of reading skill, some readers achieve higher reading rates than others.

During silent reading the average fixation lasts approximately 250 ms [1]. Hence, the assumption that this 250 ms time window represents a critical period in the word recognition process is only sensible. Due to the rapid succession of sub-processes occurring in the brain within this period, individual differences are best investigated by means of event related brain potentials (ERPs), which provide the necessary temporal resolution at a millisecond range.

The main ERP components falling within the first 250 ms after visual stimulus onset are the P1/N1 complex and the N170. Variability of the P1/N1, appearing in occipital areas, is associated with physical features of visual stimuli such as size [2] and contrast [3]. Its amplitude is also influenced by whether attention is directed to or away from a stimulus [4].

Subsequent to the P1/N1 appears a negative deflection in occipito-temporal regions, the N170, which shows sensitivity to stimulus classes (i.e., faces, words, objects) expressed in variations of hemispheric laterality, amplitude, peak latency, and habituation response [5]–[7]. In addition, participant specific N170 variance was observed for bird experts viewing birds [8] or car experts viewing cars [9]; relative to other stimulus categories experts exhibited stronger amplitudes. The function of the N170 is assumed to reflect visual structure analysis before the involvement of semantic knowledge [7]. This interpretation is supported by findings that real words compared to pseudo words [10]–[12] or unknown faces compared to famous or personally familiar faces [13], [14] do not elicit different N170 responses.

With literacy onset and its advancement, the N170 undergoes a specialization for letter strings [11], [15]. Differences in this development seem to explain reading impairments to some extent; for instance, lower N170 amplitudes for dyslexic children compared to age matched controls were interpreted as stemming from fewer neuronal circuits specialized in text processing [16]. These findings are in line with reports that even after years of text exposure, in a sample of adult non-impaired readers of German, faster readers exhibited stronger N170 amplitudes than slower readers [17].

The latter two studies reported higher amplitudes for faster readers in samples of Swiss-German and German participants, respectively. Features of the German language, especially its shallow orthography [18] might have promoted these findings. An unambiguous translation of single graphemes (i.e., letters) to phonemes, as possible in shallow orthographies, allows slow but accurate word recognition [19], [20]. Readers who developed visual recognition strategies based on letter chunks or whole words would likely benefit from speed advantages. By contrast, adult readers of Hebrew are most familiar with a script that contains almost exclusively consonant strings and does not allow unambiguous phonological decoding [21]. A diacritic system, which turns Hebrew into a completely shallow orthography, is used only in initial stages of reading acquisition [22]. Hence, adult readers of Hebrew are forced to recognize chunks of consonant letters, which might create a ceiling effect in terms of reduced variance in visual recognition strategies as compared to German readers.

Consequently, this study investigated whether also in a deep orthography such as Hebrew without diacritics, variance in visual processing would account for reading speed differences. In order to disentangle reading specific and domain general effects, we recorded ERPs while participants accomplished a lexical decision and a face decision task.

## Materials and Methods

### Ethics statement

All participants gave their written informed consent to take part in this study, which was approved by the ethics committee at the University of Haifa.

### Participants

Data for a total of 40 (19 female) participants, all university students (mean age 25.8, *SD* 3.4) and native speakers of Hebrew was collected. None of the participants reported a history of psychiatric disorders, reading or learning difficulties or attention deficit disorder. All had normal or corrected to normal vision, were right handed (according to self-report) and received monetary compensation for participation.

### Tests for reading and cognitive skills

The absence of reading impairments was verified by means of two reading tests. The One Minute Test for Words required accurately reading aloud of at least 85 unrelated words from a list per minute. A silent reading test was constructed based on principles of the Reading Speed Test (RST; F. Hutzler & H. Wimmer, personal communication, August 2006). Two parallel test forms of the Hebrew version of this test (HRST) comprise 77 short sentences, which participants had to categorize within three minutes as either meaningful or meaningless. According to an in-house norm based on a random sample of 235 university and college students the average number of sentences read in three minutes is 54.7 (*SD* = 11.2). Hence, participants had to read at least 43 sentences (i.e., 1 *SD* below the average) in order to be defined as regular readers. Using a word per minute score of the HRST, which corrected for differences of the two test forms, participants were divided in a median split into two *Reader Groups*, that is, slow and fast readers.

Five sub-tests of the Wechsler Adult Intelligence Scale (WAIS-III) [23] were used to test cognitive skills commonly associated with variance in reading capability. Similarities and Block Design served as estimates for verbal IQ and performance IQ, respectively. Digit Span (forward and backward) assessed working memory capacity; and Digit Symbol Coding as well as Symbol Search were used for testing speed of processing.

### Stimuli

Due to an initially planned training study, which demanded counterbalancing of stimuli presented before and after training, two sets of stimuli were generated for the Lexical Decision Task (LDT). For the current study participants were presented either set 1 or set 2 in equal measures. The sets were parallelized for a number of linguistic features listed in Table 1. Frequency information was taken from the word-frequency database for printed Hebrew [24]. This database and the software LINGUA [25] were used to calculate measures of bigram frequency and number of neighbors. No significant differences for any of the stimulus features were found between sets, all *t*s<1.67.

**Table 1:** Characteristics of stimuli used in the lexical decision task.

	Stimulus Set 1		Stimulus Set 2	
	*M*	*SD*	*M*	*SD*
**Word frequency** *	69.52	52.63	67.76	44.08
**Number of letters per word**	4.38	.923	4.14	.81
**Number of syllables**	2.30	.544	2.26	.57
**Mean bigram frequency words**	14753	9075	12001	7335.
**Number of neighbors words**	28.20	16.83	31.06	14.70

*appearances among 1 million according to database of Frost & Plaut [24].

Each set comprised 50 real words (nouns and adjectives) and 50 pseudo words. The pseudo words of set 1 were generated through letter replacement on the basis of real words from set 2 and vice versa. Hence each participant responded to a total of 100 stimuli.

Please note that stimuli were presented without explicit vowel information (i.e., without any diacritical dots and dashes); however, stimuli included consonant letters that could be pronounced as vowels (i.e., vav and yod) as well as characters serving as placeholders for vowels (i.e., alef and ayin).

Photographs used in the Face Decision Task (FDT) depicted 50 male and 50 female faces. Pictures were controlled for hue, contrast and saturation. None of the portrayed persons wore any makeup, piercings etc. All pictures were cropped below the neck thus not showing any clothing. 25 pictures for each gender were manipulated using the free image manipulation software GIMP. Either the nose, or an eye, or the mouth was removed by replacing these parts with adjoining skin regions.

### Task and procedure

Participants sat at a distance of approximately 60 cm in front of a computer screen in a sound attenuated room and held a joystick in their right hand. Both decision tasks (i.e., LDT and FDT) demanded pressing one of two joystick buttons (i.e., A or B) with their right thumb. During the LDT participants had to decide as quickly and as accurately as possible whether a stimulus presented for 400 ms in the middle of the screen (font: Times New Roman, 100 pixel, white on grey background) represented a real word (button A) or a pseudo word (button B). Responses were collected during the complete inter-stimulus onset period, which was set to 2100 ms. Between stimuli a blank light grey screen appeared. During the FDT participants were requested to decide again as quickly and as accurately as possible whether a photo (size 300×390 pixels, on grey background) depicted a complete face (button A) or a face with missing facial features (button B). Timing of stimulus presentation and response collection was identical to the LDT. The software Presentation (Neurobehavioral Systems) was used for stimulus presentation in both tasks. For each participant the order in which stimuli appeared was individually randomized.

### Recording procedure and ERP analysis

Using Biosemi ActiveTwo equipment (www.biosemi.com) the ongoing electroencephalogram (EEG) was recorded from 64 active Ag-AgCl pin-type electrodes mounted according to the extended 10–20 system on an elastic cap. During recording all electrodes were referenced to an active common-mode signal electrode (CMS) placed between POz and PO3. A passive driven right leg electrode (DRL) placed between POz and PO4 formed together with the CMS electrode a feedback loop representing the ground. Eye movements were monitored using three external electrodes, one pair attached to the left and right external canthi and one below the right eye. The unfiltered EEG was digitized at a 2048 Hz sampling rate.

Offline processing was conducted using the Brain Vision Analyzer 2 (Brainproducts). A bandpass filter (0.10 Hz–25 Hz, 12 dB/oct) was applied, and all electrodes were re-referenced to an average reference. Blinks and eye-movements were corrected using the method of Gratton, Coles, and Donchin [26]. After down-sampling the data to 1024 Hz, and rejecting epochs containing artifacts (e.g., channel blockings, bad gradients or excessive max–min), the continuous EEG was segmented into epochs starting 100 ms before stimulus onset and 600 ms afterwards. Average ERPs were calculated for each participant, electrode, and experimental condition, excluding trials with incorrect responses and then referred to a 100-ms pre-stimulus baseline. Only ERPs to real words and complete faces entered further analysis.

In order to estimate ERP activity for all electrodes, global field power (GFP) [27], was computed for each participant and grand averages for each condition and reader group were calculated separately. For each task, difference waves were created by subtracting data point wise the grand average ERPs of slow readers from those of faster readers. Mean amplitude around difference wave peaks was used to test whether amplitude differences observed in these time windows would differ significantly.

## Results

### Behavioral data

HRST scores were used for Reader Group allocation; hence, as expected slow readers read less words per minute (*M* = 139.88, *SD* = 14.71) than fast readers (*M* = 185.68, *SD* = 18.82), *t*(38) = −8.58, *p* = .000, Cohen’s *d* = −2.71. However, reading accuracy (i.e., the number of incorrectly categorized sentences in the HRST; *M* = 1.7, *SD* = 1.4) did not differ between reader groups, *t*(38) = −.89, *ns*.

After the elimination of outliers, defined as either above or below two standard deviations of the individual mean, reaction times (RT) for both decision tasks were averaged for correct responses. RTs to words in the LDT differed significantly between slow (*M* = 691 ms, *SD* = 84) and fast readers (*M* = 632 ms, *SD* = 78), *t*(38) = 2.29, *p* = .027, Cohen’s *d* = 0.74. The same effect was found for pseudo words, that is, slower readers showed longer RTs (*M* = 773 ms, *SD* = 80) than faster readers (*M* = 698 ms, *SD* = 75), *t*(38) = 3.05, *p* = .004, Cohen’s *d* = 0.99.

However, no Reader Group differences were found for RTs in the FDT to normal faces, *t*(38) = 1.3, *ns*, or to faces with missing features, *t*(38) = 1.61, *ns*. RT descriptives for both groups are listed in Table 2.

**Table 2:** Reaction times of the Face Decision Task and standard scores of cognitive tests for both reader groups.

	Fast Readers		Slow Readers	
	*M*	*SD*	*M*	*SD*
**RT – complete faces** 1	657	94	702	123
**RT – missing facial features** 1	632	94	679	92
**Similarities** 2	12.6	2.8	11.7	2.3
**Block Design** 2	12.2	3.4	11.9	2.1
**Digit Span** 2	12.5	3.4	12.2	3.0
**Digit Symbol Coding** 2	11.7	1.9	10.0	2.1
**Symbol Search** 2	13.0	2.8	10.0	1.8

1 in milliseconds;

2 standard scores range from 1–19, thus 10 represents an average score.

Response accuracy in both decision tasks was very high, that is, 97.10% (*SD* = 4.17) in the LDT and 96.48% (*SD* = 3.03) in the FDT, and did not differ between Reader Groups, all *t*s<1.43.

Table 2 summarizes the results of the cognitive test. Reader groups did not differ in three out of five cognitive tests, all *t*s<1.10. Only scores of Digit Symbol Coding and Symbol Search were significantly higher for faster readers, *t*(38) = −2.6, *p* = .013, Cohen’s *d* = −0.84, and *t*(38) = −4.0, *p* = .000, Cohen’s *d* = −1.30, respectively.

### ERP data

**Figure 1:**
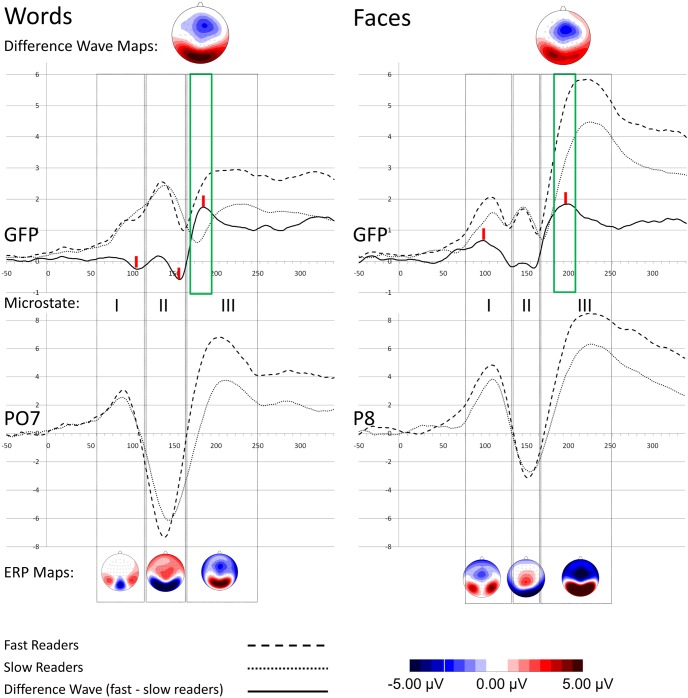
Grand average waveforms and scalp maps. The upper panel shows global field power (GFP) curves for words (left) and faces (right). ERP waveforms at selected electrode positions are shown in the lower panel. Grey squares indicate microstate boarders, red vertical dashes in the upper panel mark difference wave peaks, and the green squares indicate time window boarders where significant mean amplitude differences between reader groups were found. Maps in the upper panel show the scalp distribution of differences waves averaged over time points in the relevant time window. The maps below represent topographical maps at ERP peaks of the grand average computed across both reader groups.

Since fixation durations during reading last on average 250 ms for adults [1], ERP analyses were restricted to a time window from stimulus onset until 250 ms afterwards. Based on visual inspection so-called microstates [27] were defined around local GFP maxima. Microstate boarders were set accordingly on local minima preceding and succeeding the maxima. Consequently, three GFP peaks indicated for both decision tasks at least three distinct microstates (see Figure 1). Topography maps show that microstate I and II correspond to the ERP components P1 and N1, respectively. Microstate III represents a large component characterized by fronto-central negative activity and positive activity in occipital regions. Waveforms on electrode positions where the N170 component is conventionally measured (i.e., P07 for words and P8 for faces) show a negative peak at 140 ms for words and at 150 ms for faces, which corresponds in terms of peak latency and topographical map more to a stimulus-unspecific N1 than to a category-specific N170 component. It rather seems that the strong amplitude of the component observed in microstate III overlaps the time window, where the N170 should occur.

Further analyses focused on difference wave peaks in order to identify time windows where brain activity of slower and faster readers differed. After visual inspection of the difference waves at GFP, the three highest peaks for words (at 105 ms, 155 ms and 185 ms) and the two highest peaks for faces (at 95 ms and at 195 ms), were selected (peaks marked with red dashes in Figure 1). Then, the mean amplitude comprising 20 ms around these peaks (±10 ms) of each participant was transmitted to statistical analysis.

For both tasks, independent-sample *t*-tests comparing mean amplitude at GFP of faster and slower readers did not find significant differences in time windows before 170 ms, all *t*s <1.30. However, for the time window 175–195 ms mean amplitude was significantly higher for faster readers in the LDT, *t*(38) = −2.46, *p* = .018, Cohen’s *d* = −0.80. In a slightly later time window this effect was also visible in the FDT. Namely, between 185–205 faster readers elicited stronger amplitudes than slower readers to faces, *t*(38) = −3.32, *p* = .002, Cohen’s *d* = −1.1.

## Discussion

The results of the current experiment demonstrate that the magnitude of brain activity of slower and faster readers starts to differ significantly in time windows, which are associated with visual processing also for readers of a deep orthography (i.e., Hebrew without diacritics). This effect was found for tasks that involve the reading of words and the processing of non-linguistic stimuli (i.e., faces).

The direction of the effect is in concordance with results reported by Kast et al. [16] and Korinth et al. [17] that is, in time windows 170–190 ms after word presentation onset activity is stronger for faster readers. However, the current study could not associate this effect directly to the N170 component. ERP waveforms and maps in Figure 1 show that a clear N170, which should succeed the P1/N1 complex, is missing. Instead, a large positive component in occipital and occipto-temporal regions dominates the third microstate.

Previous research revealed that the magnitude of P1/N1 amplitudes is directly related to stimulus size [2], [3]. A possible explanation for the effect of the missing N170 might be that the relatively large stimuli in combination with a short eye-screen distance and long presentation duration of 400 ms led to a strong activation of the primary visual cortex, superimposing the N170. However, stimulus size was not manipulated systematically in this experiment, which is why this explanation remains a speculation. Further studies will have to investigate how variations in stimulus size affect the N170 component.

Nevertheless, the time course of reader group divergence represented by difference waves on GFP indicates clearly that a maximum difference between fast and slow readers was reached at 180 ms for words and 195 ms for faces, which corresponds to time windows typically showing the N170. One might argue that the choice of time windows for statistical analysis was selective and that additional peaks should have been tested. However, none of the relatively small difference wave peaks before 170 ms after stimulus onset revealed significant reader group differences, which is why it is very unlikely that reader group differences could appear on even smaller peaks. Consequently, our data does not provide reason to assume that misdirected attention allocation caused delayed word identification, since no significant amplitude differences were found in P1 or N1 time windows.

Note that relatively slow reading rates were apparently not the outcome of lower IQ or reduced working memory capacities. The only cognitive sub-skills indicating significant reader group differences compose the speed factor of the WAIS-III [23], that is, Digit Symbol Coding and Symbol Search. These subtests demand the rapid translation of non-linguistic visual patterns (i.e., not letters) into numbers and symbols and depend therefore extensively on speed and accuracy of visual processing skills. This finding provides additional support to the view that domain general visual processing skills affect reading speed.

In conclusion, the current data provide further evidence for the significance of visual processing skill as an explanatory factor of reading speed differences. This effect can’t be attributed solely to an expertise function as it was reported for car or bird experts [8], [9]. The expertise notion assumes intensive domain specific exposure to a visual stimulus class leading to stronger amplitudes in the N170 time window. An association of reading proficiency and text exposure might explain stronger amplitudes of fast readers to words, but it would not explain an amplitude increase for faces. Hence, domain-general visual processing capabilities are likely playing an important role in reading and reading acquisition.

Interestingly, this applies also for adult non-impaired readers of an orthography, which demands highly developed visual processing skills of letter chunks up to whole words as a prerequisite for successful word recognition. A ceiling effect that might have been expected for these readers was not apparent. These results contribute to our understanding of the reading process in general and might stimulate research in the field of reading impairments.

## Supporting Information

Table S1 **Characteristics of stimuli used in the Lexical Decision Task.** The table contains the complete list of items used in the LDT. For each item information is provided about its word frequency (as number of appearances per million), number of letters and syllables, which of the two stimulus sets it belongs to, summed bigram frequency, mean bigram frequency, SD of bigram frequency, number of neighbors, mean frequency of neighbors, SD of frequency neighbors, summed frequency of neighbors.
(XLSX)

Table S2 **Participant statistics and individual scores of behavioral tests.** The table provides for each participant information about age, sex, Reading Speed Test scores (words per minute, number of sentences read in three minutes, number of mistakes, which of two versions), number of words per minute read orally, response times and accuracy values for the Lexical Decision Task and the Face Decision Task, and standard scores for cognitive tests (i.e., Digit Span, Block Design, Similarities, Digit Symbol Coding, Symbol Search).
(XLSX)

Table S3 **Individual mean amplitudes values.** Each row represents one participant. Columns are labeled following the pattern: electrode position, task (LDT or FDT), condition (word or complete face), and difference peak number. For example, the column ‘CP3_LDT_word_DP1’ contains the mean activity at the CP3 electrode position elicited by words around the first difference wave peak, that is, between 95 and 115 ms after stimulus onset.
(XLSX)

Data S1 **Grand average waveforms for all electrode positions.** ERPs averaged over participants of the two reader groups and their difference waves are available here in a spreadsheet format allowing the interested reader to create waveforms for electrode positions not presented in Figure 1. Tabs at the bottom of each sheet indicate condition, reader group, or difference wave. Electrode positions are denoted in the top row. Each row represents one time frame starting from 100 ms before until 600 ms after stimulus onset.
(XLSX)
